# Mixed-Model Curriculum for Nerve Block Education in Emergency Medicine Residency

**DOI:** 10.7759/cureus.37621

**Published:** 2023-04-15

**Authors:** Samantha A King, Alexis Salerno, Kevin J Flanagan, Brian D Euerle

**Affiliations:** 1 Emergency Medicine, University of Maryland School of Medicine, Baltimore, USA

**Keywords:** point-of-care ultrasound, nerve block, ultrasound-guided nerve block, pain management, ultrasound-guided interventional pain management, e-learning, medical resident education

## Abstract

Introduction

With the rising opioid epidemic, there has been a push for multimodal pain management within the emergency department. Nerve blocks have been shown to be an effective pain management strategy for many conditions, with improved success when used with ultrasound. However, there is no generally accepted method for teaching residents how to perform nerve blocks.

Materials and methods

Seventeen residents from a single academic center were enrolled. The residents were surveyed pre-intervention regarding demographics, confidence, and use of nerve blocks. The residents then completed a mixed-model curriculum that included an electronic module (e-module) on three plane nerve blocks and a practice session. Three months later, residents were tested on their ability to independently perform the nerve blocks and resurveyed regarding confidence and use.

Results

Of the 56 residents in the program, 17 enrolled in the study; 16 participated in the first session, and nine participated in the second session. Each resident had < four ultrasound-guided nerve blocks prior to participation with a slight increase in the total number of nerve blocks after the sessions. Residents were able to perform, on average, 4.8 of seven tasks independently. Residents who completed the study reported feeling more confident in their ability to perform ultrasound-guided nerve blocks (p = 0.01) and to complete associated tasks (p < 0.01).

Conclusion

This educational model resulted in residents completing the majority of tasks independently with improved confidence in ultrasound-guided nerve blocks. There was only a slight increase in clinically performed blocks.

## Introduction

The limits of opioid analgesia, including tolerance and addiction, can be easily recognized in the treatment of patients with painful conditions in the emergency department (ED). Over the last several years, many organizations have encouraged opioid alternatives in the treatment of pain. The current American College of Emergency Physicians (ACEP) guidelines on the treatment of acute pain in the ED recommend the optimization of multimodal pain therapy strategies, including nerve blocks [1]. Nerve blocks are an effective means of analgesia for a wide variety of conditions, many of which are applicable to the ED [1-4]. Studies have shown that nerve blocks have the potential to reduce the length of ED stay and to be as effective as opioids, without the potential for sedating effects [5,6].

Fascial plane blocks are a subset of nerve blocks that allow for larger regional anesthesia. Fascia iliaca blocks, serratus anterior blocks, and transversus abdominis blocks are three types of fascial plane blocks that have been shown to be effective tools for managing pain in the ED [4,7-9]. The use of ultrasound guidance for these procedures has been shown to increase the success rates of nerve blocks and reduce complications [5,10]. However, the use of nerve blocks is still limited in the ED [10,11]. One potential reason for their lack of clinical integration is the lack of provider knowledge and limited opportunities to learn nerve block techniques [12].

One strategy to increase the use of nerve blocks in the ED is to increase provider awareness of and comfort with the procedure. Simulation has been increasingly used in medical education as it allows for teaching and procedural practice without the stress of performing the procedure on an actual patient [13]. Nerve blocks have been taught with success using simulation [14]. However, there is limited evaluation of learning through an e-learning module and more realistic simulation models, such as freshly embalmed cadavers as a means of education for emergency medicine (EM) residents.

The purpose of this study is to assess the effectiveness of teaching EM residents nerve block techniques via a mixed-model educational curriculum with e-learning modules and cadaveric sessions as determined by survey completion, procedural performance, and assessment of image acquisition after completion of the training.

## Materials and methods

Participants and recruitment

This was a prospective study of a convenience sample of EM residents at a large urban academic medical center. This study was approved by the University of Maryland, Baltimore Institutional Review Board (HP-00096894). We invited 56 current residents in the categorical EM, combined emergency medicine/internal medicine (EM/IM), and combined emergency medicine/pediatrics (EM/Peds) residency programs to participate in the study. In-person sessions took place at the Maryland State Anatomy Board cadaver lab during a cadaveric session, which is an existing part of the residency education curriculum. Residents were recruited via email, where they were given study information and completed a written consent form. Upon return of the completed consent form, residents were given access to the e-module and further instructions for cadaver lab sessions. Residents who declined to participate were excluded.

Those who agreed to participate were asked to (1) complete an online pre-participation survey (Appendix A); (2) complete an e-module reviewing fascial plane nerve blocks; (3) participate in an in-person cadaveric session and practice three nerve blocks; (4) perform three nerve blocks at a second in-person cadaveric session three months later; (5) complete a post-participation survey.

Pre-participation and post-participation survey

Residents were asked to complete a questionnaire prior to completing subsequent steps in the study and at the end of their participation in the study. Residents participating in the study provided demographic information regarding their program (EM, EM/IM, or EM/Peds) and current post-graduate level year. Residents were asked to estimate the number of nerve blocks and ultrasound-guided nerve blocks they had completed at the respective points in the study. The responses were then grouped into ranges of 0, 1-3, 4-6, 7-9, and >10. The final set of questions asked residents to rate their comfort level with performing nerve blocks, materials and medications needed for ultrasound-guided nerve blocks, and performance of ultrasound-guided nerve blocks prior to completing the e-module and after the second cadaveric session. The questions used a five-point Likert scale with 1 = not confident at all and 5 = proficient and independently practicing.

Online e-module

After completion of the pre-participation survey, residents were given access to the e-module, which remained available to them for the duration of the study. The e-module was created using Articulate360 (Articulate Global LLC, New York, NY) and stored on the residency program’s Moodle platform (Moodle^TM^ 3.11, https://moodle.org; West Perth, Australia). The outline of the module followed the principles of the I-AIM (Indication, Acquisition, Interpretation, Medical Decision-making) model [15]. The e-module taught residents the indications and techniques for fascia iliaca, serratus anterior, and transversus abdominis fascial plane blocks. After the final cadaveric lab session, the e-module was made available to the entire residency program.

Cadaveric teaching session

After completion of the pre-participation survey and e-module, residents attended a cadaveric simulation session as a part of their residency curricula. Cadavers for these sessions were prepared via arterially flushing with a preservative solution and stored in 40°F containers. During this session, residents who had enrolled in the study were given individualized instruction on techniques for the three nerve blocks on an available cadaver. Each resident was given the opportunity to complete the three nerve blocks at least once during their instruction time. No data regarding their performance were collected during this session. Nerve blocks on the cadavers were completed using 20-gauge Quincke-type spinal needles (Becton, Dickinson and Company, Franklin Lakes, NJ), a j-loop connector piece, and a syringe filled with tap water. Spinal needles were used in this study because commercial nerve block kits are more expensive and may not be available in all EDs. Images were obtained with a Butterfly iQ+ (Butterfly Network Inc., Guilford, CT) and displayed on an iPad Pro (Apple Inc., Cupertino, CA).

Cadaveric examination session

During the next scheduled cadaveric laboratory and simulation session, three months after the prior, residents who had completed the above steps were asked to complete the three nerve blocks with the grading of technique. Each resident was assessed individually on their ability to complete the three nerve blocks. For each nerve block, the resident was graded on their ability to complete a series of seven steps, which would result in the successful completion of the nerve block (Appendix B) [15-17]. Residents were randomly assigned, using a dice roll, an order of nerve blocks to complete. Nerve blocks on the cadavers were completed using the same procedure as described above. At each step, if the resident was unable to complete a step in the process, they were given instructions on how to complete that step, prior to proceeding to the next step. Residents were given a point for each of the seven steps completed without receiving additional instruction during that step. Residents were then given a final score out of seven. After completion of the nerve blocks, residents were given the opportunity to ask questions regarding their performance and regarding the nerve block instruction. Residents then completed a post-participation survey as noted above.

Statistics

Demographic data and answers to the number of nerve blocks previously completed were tabulated. Simple descriptive statistics were used to analyze the data. The primary objective of this study was to evaluate the resident performance of fascial plane nerve blocks after the completion of the e-module and instructional session. Residents' scores were averaged for all blocks completed, each block type completed, and block number completed. Standard deviations were calculated using Microsoft Excel 2022 (Microsoft Corporation, Redmond, WA). Wilcoxon signed-rank tests were performed to evaluate the pre- and post-participation questionnaires.

The secondary objective of this study was to assess the confidence of residents to complete the ultrasound-guided nerve blocks. The average score and standard deviation for each Likert-scale question were calculated. Wilcoxian signed rank tests were performed to compare pre-participation and post-participation responses.

## Results

Demographics

A total of 17 residents out of 56 total combined and categorical EM residents consented to participate in the study. Of the 17 who initially enrolled, 13 residents (76%) completed the first survey, 16 (94%) participated in the first educational day, and a total of nine participants (53%) participated in the second cadaveric session and completed the second survey (Table 1) with the majority of survey completions being categorical EM residents. Most participants had completed fewer than six nerve blocks prior to the initiation of the study and only 15% reported having completed a nerve block under ultrasound guidance. After completion of the study, 33% reported completion of an ultrasound-guided nerve block (Table 1).

**Table 1:** Demographics of participants in pre-participation and post-participation surveys.

Survey question		Pre	Post
Type of program			
	Emergency medicine (EM)	7	8
	Emergency medicine/internal medicine (EM/IM)	5	1
	Emergency medicine/pediatrics (EM/Peds)	1	0
Year			
	1	6	3
	2	3	2
	3	3	2
	4	1	0
# of nerve blocks completed			
	0	3	0
	1-3	3	2
	4-6	3	3
	7-9	1	0
	10+	2	2
# of nerve blocks with ultrasound completed			
	0	11	5
	1-3	2	1
	4-6	0	2
	7-9	0	0
	10+	0	0

Surveys

Prior to the study, participants completed a survey on comfort level with nerve blocks and filled out the survey again post-participation. Prior to completion of any education, residents ranked the majority of questions regarding their comfort level with ultrasound and nerve blocks as less than 3 out of 5; however, after the study, there was an overall general increase in their comfort level. There was an increase in modified comfort levels for residents completing the survey regarding the performance of and obtaining appropriate images of ultrasound-guided nerve blocks (Table 2). There was also an increase in resident comfort with obtaining supplies for and choosing the correct medications for ultrasound-guided nerve blocks. No difference was found in resident comfort for the performance of nerve blocks in general, using ultrasound or identifying musculoskeletal structures on ultrasound. Of note, no resident answered any of the questions with a 5 (highest comfort level) in either survey.

**Table 2:** Average score and standard deviation for resident comfort performing selected tasks for ultrasound-guided nerve blocks before and after educational sessions. Scale of 1 to 5 (1 = non-confident, 5 = independently practicing).

Question	Pre	Post	P-value	
Performing nerve blocks?	1.8	2.4	0.08	
Performing ultrasound-guided nerve block?	1.5	2.3	0.02	
Obtaining supplies for a nerve block?	2.1	3.2	0.004	
Choosing the correct medications for a nerve block?	2.1	3.4	0.001	
Using ultrasound in clinical practice?	3.1	3.8	0.1	
Identifying musculoskeletal structures on ultrasound?	2.3	3.0	0.1	
Obtaining images for nerve blocks on ultrasound?	1.7	2.8	0.02	

Nerve block technique assessment

Residents were graded by ultrasound faculty on seven core procedural tasks in completing nerve blocks during the second cadaveric lab day. Residents were able to complete on average 4.8 of the seven tasks correctly without instruction (Table 3).

**Table 3:** Proportion of residents completing each core task correctly during the second educational session.

Task	Average task completion
Identify anatomical landmarks of the area of interest	0.69
Identify anatomical landmarks of the area of interest on ultrasound	0.81
Identify the area of injection on ultrasound	0.69
Inserts the needle at the correct angle	0.65
Visualizes needle under ultrasound	0.73
Inserts needle to correct depth	0.58
Injects "anesthetic" into the correct plane	0.65
Total	4.81

**Figure 1:**
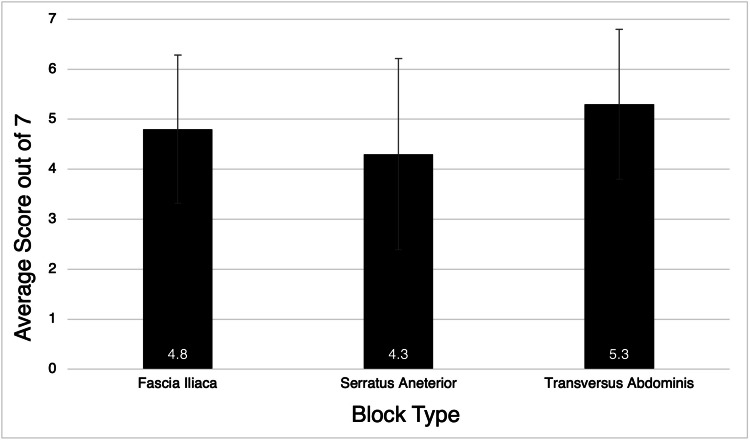
Average number of correct tasks completed by residents during the second educational session by nerve block type. Error bars indicate standard deviations.

Residents had the most difficulty with the insertion of the needle to the correct depth. There was no significant difference in the total number of tasks completed correctly for each type of block (Figure 1).

**Figure 2:**
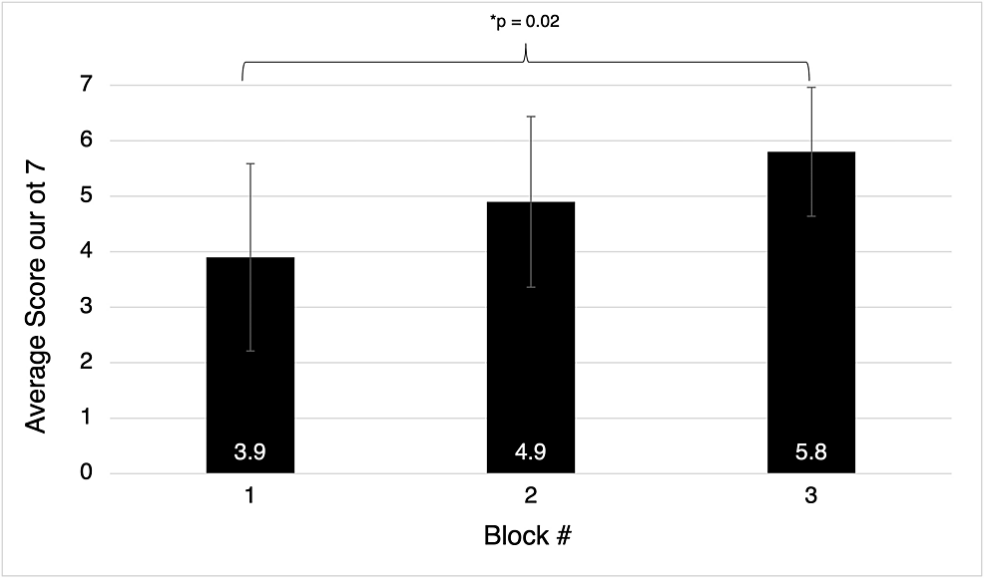
Average number of correct tasks completed by residents during the second educational session by nerve block number completed. Error bars indicate standard deviations.

However, 100% of participants on the second cadaver day were able to identify relevant anatomy for the fascia iliaca block, more than the other two blocks (50% for serratus anterior and 89% for transversus abdominis blocks) but struggled the most with correct needle insertion depth for this block with only 44% completing it without assistance. In comparing the proportion of correctly completed tasks for each consecutive block, there was a significant increase in the total proportion of tasks performed correctly from the first block completed (3.89 tasks on average) to the third block completed (5.75 tasks on average, p = 0.02) (Figure 2).

## Discussion

The overall goal of this study was to create a mixed-model curriculum for teaching nerve blocks to EM residents. For this study, an online survey and an e-module were created for residents to complete prior to in-person teaching sessions. Residents were then taught three nerve blocks during an in-person cadaveric session held during pre-scheduled educational sessions. These sessions followed the I-AIM method for ultrasound-guided scanning procedures [18]. They were then evaluated three months later on their ability to perform the nerve blocks. To our knowledge, there are no prior reports of a teaching intervention for a diverse set of nerve blocks, rather than one type of nerve block.

For a Kirkpatrick level 1 training evaluation, we asked residents to complete a survey about their confidence in the procedure [19,20]. Residents who completed this study reported a significant increase in their confidence to complete tasks surrounding ultrasound-guided nerve blocks. Residents felt more confident in their ability to obtain materials, identify correct medications, obtain images of their nerve block, and in their overall ability to perform an ultrasound-guided nerve block. These results would suggest that the study had a positive impact on their education. In contrast, residents did not report a significant increase in their ability to identify musculoskeletal structures on ultrasound or in their confidence in using ultrasound clinically. It is possible that continued exposure and more frequent teaching of ultrasound may have resulted in a larger positive impact [21].

For a Kirkpatrick level 2 training evaluation, we asked residents to perform the three learned nerve blocks [19,20]. Residents were only able to successfully complete just over half of the seven steps successfully without instruction during the second session, likely attributed to overall limited exposure to nerve blocks between the teaching sessions. However, throughout the intervention, residents showed a general trend of increased competency with each nerve block type, regardless of the order. Skill repetition alongside expert intermittent feedback has been shown to result in improved student performance [21]. The residents in our study might have benefited from more frequent intervention and skill repetition. It has been proposed that a refresher course every six to 12 weeks may be beneficial for maintaining and improving complex skills [22].

For a Kirkpatrick level 3 training evaluation, we surveyed the residents on their clinical practice after the training session [19,20]. There was only a slight trend upward in the number of ultrasound-guided nerve blocks completed before and after the study, suggesting that despite education, there was minimal incorporation of the procedure into clinical practice. This lack of increase may be related to overall limited use in clinical practice by attendings at this institution, similar to the general practice of EM [11,12]. Lack of use in clinical practice may have contributed in part to at least some skill degradation and therefore lack of observed improvement in the residents. More frequent teaching sessions or exposures may have led to overall improved scores in the second session. As seen in other simulation studies, this study was unable to assess the impact on patient outcomes, i.e., level 4 of the Kirkpatrick training evaluation model [19]. Given the lack of change in the number of ultrasound-guided nerve blocks performed between the two segments, there would be little expected change in patient outcomes.

Other educators have attempted to develop nerve block education models through the use of simulation [23-25]. Sparks et al. showed that a porcine model could be created as a means of teaching general nerve block technique [14]. Limited prior studies have shown that cadavers can be an appropriate simulation model for the teaching of fascia iliaca nerve blocks [26]. However, none of these studies evaluated the teaching of the technique and maintenance of the skill over time. Our study showed that many residents maintained some amount of skill at the testing date and were able to complete four out of seven of the steps during the simulation. Some residents did receive mid-session teaching if the resident did not remember how to perform the required step and requested assistance. In the future, it would be valuable to use similar training programs for other medical specialties.

Limitations

This study was performed at a single urban academic residency program that may not be representative of emergency medicine residency programs throughout the country. Only a small proportion of residents were able to participate in the study. This limitation was likely due to the inability of residents to attend after an overnight or after a 24-hour call. Additionally, combined residents made up a fair proportion of the participants in the course who alternate between emergency medicine rotations and their combined program rotations, limiting the number who had time off for the second session. Finally, given the residents’ limited exposure to nerve blocks prior to this educational session, they were not evaluated during the initial session, limiting the data available for a fuller assessment of skills acquisition and degradation.

## Conclusions

Ultrasound-guided nerve blocks serve as a part of multimodal pain management in the ED. Our study suggests that residents can be taught how to perform nerve blocks and can maintain some of these skills over time. However, learners may need additional education and experience with performing nerve blocks prior to integrating them into their clinical practice.

## Appendices

Appendix A

Nerve Block Cadaver Simulation Study Survey

1. Did you complete the consent form? (Hard stop, cannot proceed without selecting yes)

Yes

No

2. What type of resident are you (Select one)?

Emergency Medicine

Emergency Medicine/Internal Medicine

Emergency Medicine/Pediatrics

3. What year in residency are you?

4. How many nerve blocks (including digital, plane blocks, etc.) have you performed?

5. How many ultrasound-guided nerve blocks have you performed?

6. How comfortable are you in performing nerve blocks? (1 - not confident at all, 5 - proficient and independently practicing)

7. How comfortable are you in performing ultrasound-guided nerve blocks? (1 - not confident at all, 5 - proficient and independently practicing)

8. How comfortable are you with obtaining supplies to perform a nerve block? (1 - not confident at all, 5 - proficient and independently practicing)

9. How comfortable are you with selecting appropriate medications for nerve blocks? (1 - not confident at all, 5 - proficient and independently practicing)

10. How comfortable are you with using ultrasound in your clinical practice? (1 - not confident at all, 5 - proficient and independently practicing)

11. How comfortable are you with identifying musculoskeletal structures on ultrasound? (1 - not confident at all, 5 - proficient and independently practicing)

12. How comfortable are you with obtaining appropriate images for the completion of nerve blocks using ultrasound? (1 - not confident at all, 5 - proficient and independently practicing)

Appendix B

Nerve Block Acquisition Grading Sheet

**Table 4:** Nerve block acquisition grading sheet

Which nerve block (circle)?	Fascia iliaca, transversus abdominis, and serratus anterior
Task	Completed correctly (Yes/No)
Identify anatomical landmarks of the area of interest (prompted). Fascia iliaca: inguinal ligament, area of vasculature. Transversus abdominis: lateral abdominal wall. Serratus anterior: pectoralis muscle, latissimus dorsi muscle, mid-axillary line	
Identify anatomical landmarks of the area of interest on ultrasound (prompted). Fascia iliaca: femoral artery and vein, femoral nerve, fascia lata & fascia iliaca. Transversus abdominis: external oblique muscle, internal oblique muscle, transverse abdominis muscle. Serratus anterior: serratus anterior muscle, latissimus dorsi muscle, rib	
Identify the area of injection on ultrasound (prompted). Fascia iliaca: fascia iliaca plane. Transversus abdominis: transverse abdominis/external oblique plane. Serratus anterior: serratus anterior muscle and latissimus dorsi muscle plane	
Inserts needle at the correct angle	
Visualizes needle under ultrasound	
Inserts needle to correct depth	
Injects “anesthetic” into the correct plane	
